# Are pragmatism and ethical protections in clinical trials a zero-sum game?

**DOI:** 10.1177/17407745241284798

**Published:** 2024-10-15

**Authors:** Hayden P Nix, Charles Weijer, Monica Taljaard

**Affiliations:** 1 Department of Medicine, Dalhousie University, Halifax, NS, Canada; 2 Department of Medicine, Western University, London, ON, Canada; 3 Department of Philosophy, Western University, London, ON, Canada; 4 Department of Epidemiology & Biostatistics, Western University, London, ON, Canada; 5 Clinical Epidemiology Program, Ottawa Hospital Research Institute, Ottawa, ON, Canada; 6 School of Epidemiology and Public Health, University of Ottawa, Ottawa, ON, Canada

**Keywords:** Pragmatic trials, research ethics, vulnerability, geriatrics, critical care, psychiatry

## Abstract

**Background:**

Randomized controlled trials with pragmatic intent aim to generate evidence that directly informs clinical decisions. Some have argued that the ethical protection of informed consent can be in tension with the goals of pragmatism. But the impact of other ethical protections on trial pragmatism has yet to be explored.

**Purpose:**

In this article, we analyze the relationship between additional ethical protections for vulnerable participants and the degree of pragmatism within the PRagmatic Explanatory Continuum Indicator Summary-2 (PRECIS-2) domains of trial design.

**Methods:**

We analyze three example trials with pragmatic intent that include vulnerable participants.

**Conclusion:**

The relationship between ethical protections and trial pragmatism is complex. In some cases, additional ethical protections for vulnerable participants can promote the pragmatism of some of the PRECIS-2 domains of trial design. When designing trials with pragmatic intent, researchers ought to look for opportunities wherein ethical protections enhance the degree of pragmatism.

## Background

Schwartz and Lellouch developed the concepts of “pragmatic” and “explanatory” to capture two dichotomous intentions of clinical trial design.^1^ A trial designed with pragmatic intent aims to generate evidence that directly informs clinical or policy decisions, while a trial designed with explanatory intent aims to generate evidence about the mechanism of an intervention.

But, as the PRagmatic Explanatory Continuum Indicator Summary-2 (PRECIS-2) tool makes explicit, an investigator’s binary intention manifests in a multidimensional continuum, consisting of nine domains of trial design: eligibility, recruitment, setting, organization, flexibility (delivery), flexibility (adherence), follow-up, primary outcome, and primary analysis.^2^

The pragmatism of each domain of trial design is a function of the trial’s fidelity to how the study intervention is intended to be used in practice. Design features that increase the pragmatism of a trial include (1) eligibility criteria that are consistent with the patient population targeted by the intervention, (2) recruitment processes that seek participants from multiple usual care settings as prospective participants present to those settings, (3) conducting the trial in the setting of intended use for the intervention, (4) organizing the trial such that the study intervention is delivered using only resources that are available in usual care, (5) allowing providers to deliver the intervention with as much flexibility as they would have in practice, (6) enforcing adherence to the intervention using only measures that would be in place in usual care, (7) intensity of follow-up that is consistent with usual care, (8) outcomes that are clinically relevant, and (9) intention-to-treat data analysis.^2^

Some argue that ethical protections can be in tension with pragmatism. This literature is focused on the relationship between trial pragmatism and informed consent. Ford and Norrie correctly identify that informed consent hinders pragmatism within the domain of trial recruitment because it impedes “unselected participant recruitment,” thereby potentially rendering the pool of participants dissimilar to the study intervention’s target population.^3^ Similarly, others argue that informed consent hinders pragmatism by disrupting “the ordinary workflow of busy [clinical settings],”^4^ and increasing “the administrative costs and burdens of a study.”^5^

We believe that focusing on the impact of informed consent on trial recruitment is too narrow. Manifold ethical protections—in addition to informed consent—are relevant in trials with pragmatic intent. And the pragmatism of other PRECIS-2 domains of trial design may be impacted by ethical protections.

The analysis of the relationship between ethical protections and trial pragmatism ought to include additional ethical protections for vulnerable participants. Vulnerable participants are at “an identifiably increased likelihood of incurring additional or greater wrong,” and are entitled to additional ethical protections.^6,7^ Pragmatic trials are more likely to include vulnerable participants than explanatory trials because pragmatic trials commonly have broad eligibility criteria.^8,9^

In 2015, Welch and colleagues analyzed ethical and regulatory issues that arise from the inclusion of vulnerable participants in pragmatic trials.^9^ Their approach to navigating tensions between additional ethical protections and trial pragmatism was to re-evaluate the need for ethical protections in pragmatic trials. They argued that “not all safeguards for vulnerable subjects may be appropriate, feasible, or ethical in [pragmatic trials]” because common features of pragmatic trials, including “randomization at the group level, reliance on electronic health record (EHR) data, the availability of safety data, and comparisons of approved forms of medical care, may contribute to some [pragmatic trials] meeting the criteria for minimal risk to human research subjects.”^9^

A more detailed analysis of the relationship between additional ethical protections for vulnerable participants and trial pragmatism is warranted for two reasons. First, the need for ethical protections in pragmatic trials may be greater than Welch and colleagues suggest. A recent systematic review of 1988 pragmatic trials found that 85.0% reported obtaining informed consent, and only 1.1% reported that the study involved minimal risk.^10^ And second, a trial with pragmatic intent is socially valuable to the extent that the evidence it generates usefully informs a clinical or policy decision. Trials with highly pragmatic design features are more likely to achieve this goal. If—like informed consent—additional ethical protections for vulnerable participants reduce the pragmatism of the domains of trial design, then they may also reduce the social value of a trial with pragmatic intent.

In this article, we explore the relationship between additional ethical protections for vulnerable participants and the pragmatism of the PRECIS-2 domains of trial design by analyzing three example trials with pragmatic intent. We present PRECIS-2 diagrams, completed with input from trial principal investigators, to illustrate how alternative approaches to mitigate a specific vulnerability can impact pragmatism. We argue that the relationship between trial pragmatism and ethical protections is complex. Some additional protections for vulnerable participants can hinder the pragmatism of some domains of trial design, while others can promote the pragmatism of some domains of trial design.

## Example 1: PRiVII Trial

The Preventing Respiratory Viral Illness Invisibly (PRiVII) Trial (Table 1) is a cluster randomized trial with pragmatic intent that aims to evaluate the effect of far-UVC (222 nm) light devices on the incidence of viral respiratory infections in long-term care facilities.^11^ Discrete living areas within long-term care facilities were randomized to have small, smoke-detector size devices installed in hallways and common areas. These devices were programmed to emit either far-UVC light or standard fluorescent light in hallways and common areas. Participants exhibiting symptoms suggestive of viral respiratory infection were tested. As the study involves a cluster-level intervention that poses only minimal risk, a waiver of consent was obtained for the study intervention. Consent was sought for data collection. Cognitive impairment is prevalent in the included long-term care facilities, raising questions about prospective participants’ decision-making capacity. In lieu of conducting formal capacity assessments for the study, researchers referred to records of clinical decision-making capacity. For participants who lacked capacity, assent and surrogate consent for data collection were sought.

**Table 1.** PRiVII Trial.^11^

*Aim*: Evaluate the effect of far-UVC light (222 nm) devices in common areas in long-term care facilities on the incidence of respiratory viral illness. *Intervention*: Far-UVC light devices emitting 222 nm light installed on the ceiling in hallways and common rooms in long-term care facilities. *Control*: Devices identical to the far-UVC lights emitting ambient light installed on the ceiling in hallways and common rooms in long-term care facilities. *Data collection*: Researchers collected data from medical records. Consistent with routine care, long-term care facility staff conducted routine physical examinations and obtained nasopharyngeal viral antigen swabs as indicated. *Results*: Pending. *Consent procedures*: A waiver of consent was obtained for the study intervention because it is a minimal risk cluster-level intervention. Researchers use records of clinical decision-making capacity in lieu of formal capacity assessments. For participants who lacked capacity, assent and surrogate consent are obtained for data collection.

### Vulnerability

The PRiVII Trial was conducted in long-term care facilities. In this setting, many prospective participants had cognitive impairments.^11^ These cognitive impairments render prospective participants vulnerable to inadequate comprehension in informed consent discussions.

### Additional ethical protections

In the PRiVII Trial, researchers had several options to mitigate this vulnerability. They could have (1) conducted rigorous capacity assessments designed for clinical research (e.g. the MacArthur Competence Assessment Tool for Clinical Research);^12^ (2) conducted abbreviated capacity assessment (e.g. Palmer and colleagues’ three questions: What is the purpose of the study? What are the risks? and What are the benefits?);^13^ or (3) inferred decision-making capacity for study participation based on documented capacity for clinical decisions in prospective participants’ medical records. It is widely accepted that when a prospective participant lacks decision-making capacity to consent to trial participation, researchers generally ought to seek surrogate consent.^7^

### Impact of additional ethical protections on pragmatism

**Figure 1.**
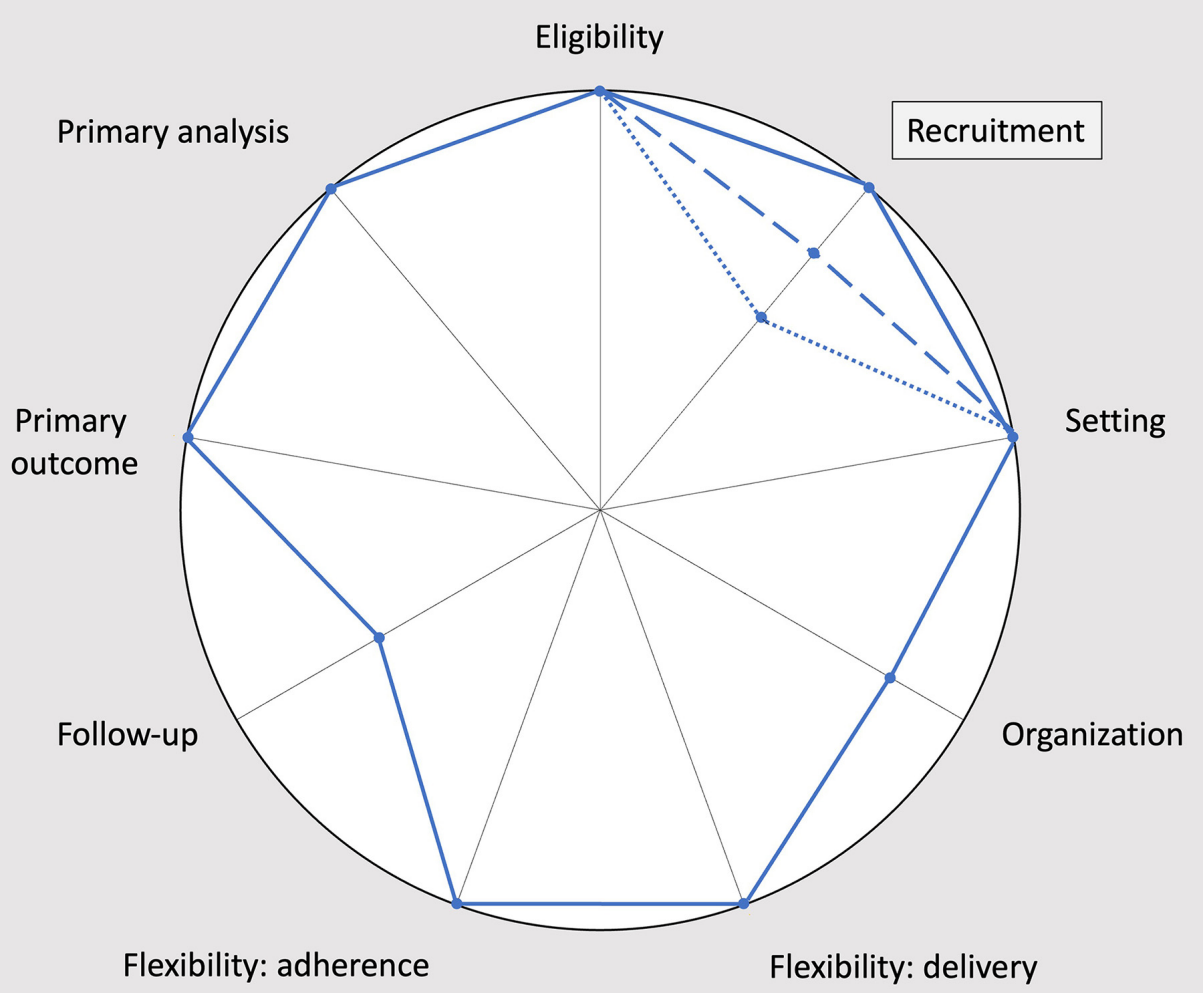
PRECIS-2 diagrams of the PRiVII Trial, corresponding to rigorous capacity assessments (dotted line), brief capacity assessments (dashed line), and using records of clinical decision-making capacity (solid line). This figure is based on the PRECIS-2 diagram in the published trial protocol.^11^

Each of these ethical protections has a different impact on pragmatism within the recruitment domain of trial design (Figure 1).

Rigorous capacity assessments would require resources to train personnel to ensure that the assessments are conducted reliably. Conducting such resource-intensive capacity assessment could incentivize researchers to minimize resource use by selectively approaching potential participants who seem to either clearly have or clearly lack decision-making capacity. If this incentive leads researchers to avoid approaching potential participants with marginal decision-making capacity, then it would bias the sample and thereby hinder the pragmatism of trial recruitment. Abbreviated capacity assessments hinder the pragmatism of trial recruitment for similar reasons, but to a lesser extent. However, abbreviated capacity assessments are less reliable than rigorous capacity assessments.

Inferring decision-making capacity for study participation based on capacity for clinical decision-making is one way to alleviate this tension. In the PRiVII Trial, a waiver of consent was obtained for the study intervention because it was a cluster-level intervention that posed only minimal risk. Only consent for data collection (i.e. routine physical exams and nasopharyngeal swabs) was required. This method of capacity of assessment was ethically permissible because the data collection procedures were akin to routine care.^14^ Checking records of clinical decision-making capacity requires few resources and does not create an incentive to avoid approaching participants with marginal decision-making capacity. Therefore, it promotes the pragmatism of trial recruitment.

## Example 2: Chlorhexidine Bath Trial

The Chlorhexidine Bath Trial (Table 2) is a cluster randomized trial with pragmatic intent that aimed to evaluate the effect of daily chlorhexidine baths on the incidence of healthcare-associated infections in patients admitted to intensive care units.^15^ Five intensive care units, including the cardiovascular, medical, neurological, surgical and trauma intensive care units, at a tertiary care medical center were randomized such that all patients in each unit received a once daily bath with either a 2% chlorhexidine solution or no antimicrobial agent. The incidence of healthcare-associated infections was collected from electronic medical records.

**Table 2.** Chlorhexidine Bath Trial.^15^

*Aim*: Evaluate the effect of daily chlorhexidine bathing of patients in intensive care units on the incidence of healthcare-associated infections. *Intervention*: Once-daily bathing with disposable cloths soaked in 2% chlorhexidine solution. *Control*: Once-daily bathing without antimicrobial agents. *Data collection*: Incidence of healthcare-associated infections were collected from electronic medical records. *Results*: Relative to daily bathing without antimicrobials, daily bathing with 2% chlorhexidine solution did not reduce the incidence of hospital-associated infections. *Consent procedures*: A waiver of informed consent was obtained for trial participation.

### Vulnerability

In the Chlorhexidine Bath Trial, patients who underwent cardiac surgery were vulnerable to a welfare wrong because they were at increased risk of healthcare-associated infections, relative to other trial participants.^15^ Usually, patients undergoing cardiac surgery receive a chlorhexidine bath immediately before their operation to decrease the risk of infection.^16^ If these patients were randomized to the control arm, then participating in the trial could have precluded them from receiving the usual care pre-operation chlorhexidine bath. For these patients, the risks of study participation might have outweighed the potential benefits, constituting a welfare wrong.

### Additional ethical protections

To mitigate this vulnerability, researchers could have (1) excluded cardiac surgery patients from the trial, or (2) had nursing staff provide cardiac surgery patients randomized to the control arm with a chlorhexidine bath immediately pre-operation, as per usual care.

### Impact of additional ethical protections on pragmatism

**Figure 2.**
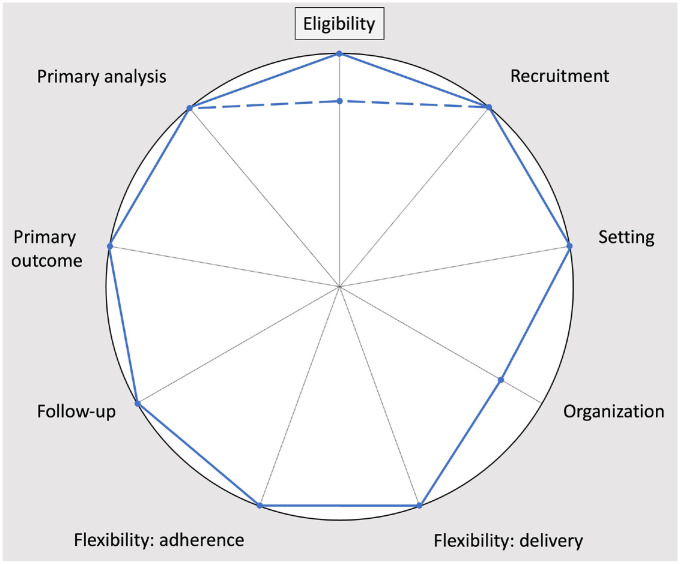
PRECIS-2 diagrams of the Chlorhexidine Bath Trial, corresponding to exclusion of surgical patients (dashed line) and inclusion of surgical patients with additional chlorhexidine baths provided by nursing staff (solid line). This PRECIS-2 diagram was verified by the Principal Investigator of the Chlorhexidine Bath Trial.^15^

Each of these additional ethical protections has a different impact on the pragmatism of the domains of trial design (Figure 2).

Excluding cardiac surgery patients from the trial would effectively mitigate the vulnerability because it would have allowed these patients to receive usual care, undisrupted by study procedures. However, cardiac surgery patients are a relevant patient population for this trial. Cardiovascular and surgical intensive care units were included in the study, demonstrating that the researchers intended the results to generalize to cardiac surgery patients. Furthermore, cardiac surgery patients are at high risk of healthcare-associated infections, making the study particularly socially valuable for this group.^16^ Therefore, removing this patient population from the pool of participants would reduce the pragmatism in the domain of trial eligibility.

In the Chlorhexidine Bath Trial, nurses provided cardiac surgery patients randomized to the control arm with a chlorhexidine bath immediately before surgery. This additional protection mitigated the increased risk of infection. It also promoted the pragmatism of trial eligibility by including a part of the clinical population to whom the intervention would be delivered in practice. Furthermore, it promoted the pragmatism of trial organization because the additional protection was delivered by personnel who are present in usual care.

## Example 3: ED to EPI Trial

The Emergency Department to Early Psychosis Intervention (ED to EPI) Trial (Table 3) is a randomized controlled trial with pragmatic intent that aimed to evaluate the effect of a text message reminder intervention on attendance at Early Psychosis Intervention programs.^17^ Patients presenting to the emergency department with psychosis were randomized to receive either a series of text messages (appointment reminders, educational material, and check-ins to rate distress) or a single text message (notification that they would be contacted to book an appointment). Participates who lacked a cell phone were provided with a prepaid cell phone at the time of enrolment. Records of participation in the Early Psychosis Intervention program were collected from electronic medical records.

**Table 3.** ED to EPI Trial.^17^

*Aim*: Evaluate the effect of a text message intervention on attendance at Early Pschyosis Intervention programs on young patients presenting to emergency departments with psychosis. *Intervention*: A series of text messages (appointment reminders, psychoeducational material, and check-ins to rate distress) sent to patients awaiting psychiatric follow-up for psychosis. *Control*: A single text message, sent shortly after enrolment, sent to notify patients that they would be contacted to book an appointment for the Early Psychosis Intervention program. *Data collection*: Records of participation in the Early Psychosis Intervention program are collected from electronic medical records. *Results*: Pending. *Consent procedures*: Researchers seek consent from prospective participants in emergency departments. Electronic consent is sought from eligible participants who present to the emergency department outside business hours via text message or email.

### Vulnerability

In the ED to EPI Trial, patients who lack cell phones are vulnerable to unjust exclusion. A pilot study that preceded the ED to EPI Trial found that less than 5% of prospective participants lacked a cell phone.^17^ This group is at high risk of being lost to follow-up for psychiatric care, making the ED to EPI Trial particularly socially valuable. Excluding patients who lack cell phones would limit the generalizability of the results of the trial and thereby potentially deprive this group of an evidence-based intervention.

### Additional ethical protections

Researchers had two options to address this vulnerability: (1) exclude patients who lack cell phones or (2) provide patients who lack cell phones with prepaid cell phones.

### Impact of additional ethical protections on pragmatism

**Figure 3.**
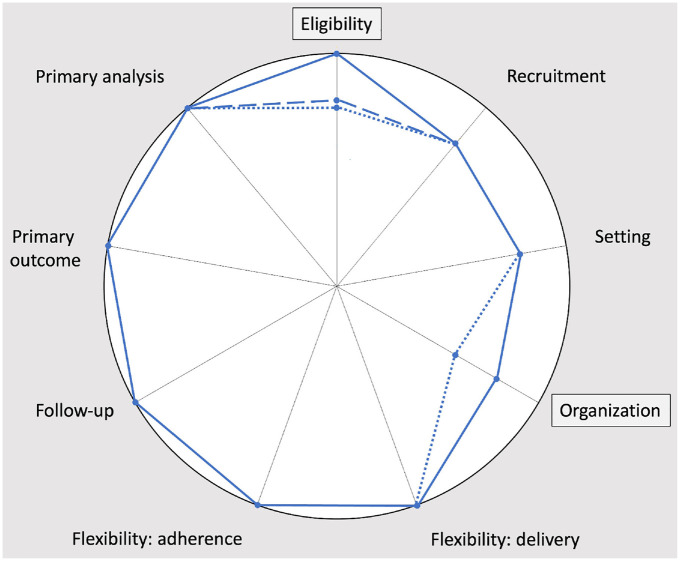
PRECIS-2 diagrams of the ED to EPI Trial, corresponding to prepaid cell phones not used in the trial but used in practice (dashed line); prepaid cell phones used in the trial but not in practice (dotted line); and prepaid cell phones used in the trial and in practice (solid line). This PRECIS-2 diagram was verified by the Principal Investigator of the ED to EPI Trial.^17^

The impact of prepaid cell phones on the pragmatism of the domains of trial design hinges on whether researchers intend for the intervention to (1) target people who lack cell phones and (2) include prepaid cell phones when it is implemented in practice (Figure 3).

If the intervention was not meant to target people who lack cell phones and prepaid cell phones were not meant to be part of the intervention in practice, then this additional protection would reduce the pragmatism of trial eligibility by including a group who would not be able to receive the intervention in practice. Furthermore, delivering the intervention in the trial using financial resources that will not be present in practice would reduce the pragmatism of trial organization.

However, in the ED to EPI Trial, researchers intended for the intervention to target people who lack cell phones. They obtained additional funding for prepaid cell phones to facilitate the inclusion of this group. For the intervention to be effective for this group in practice, it must include prepaid cell phones. Therefore, prepaid cell phones promote the pragmatism of trial eligibility and trial organization by facilitating the inclusion of a target patient population using resources that are meant to be present in usual care.

## Discussion

Many have argued that standard informed consent requirements are in tension with the pragmatism of trial recruitment. But few have considered the impact of other additional protections on trial pragmatism.

Analyzing the impact of additional ethical protections for vulnerable participants on the pragmatism of the domains of trial design reveals a more complex relationship between ethical protections and trial pragmatism. We have demonstrated that additional ethical protections for vulnerabilities to autonomy, welfare, and justice wrongs can, in fact, have positive impacts on the pragmatism of trial recruitment, eligibility, and organization. Some ethical protections can hinder the degree of pragmatism within some domains of trial design. Other ethical protections can promote the degree of pragmatism in some domains.

To mitigate a vulnerability in a trial, investigators often have an array of additional ethical protections from which to choose. Investigators designing trials with pragmatic intent that include vulnerable participants ought to consider a range of additional ethical protections for each vulnerability and evaluate the impact of each option on the pragmatism of the domains of trial design. When all else is equal, they ought to adopt ethical protections that promote, rather than hinder pragmatism.

When tension between an ethical protection and the pragmatism of a domain of trial design is irresolvable, we believe ethical protections should be prioritized. Ethical protections are essential for protecting the rights and welfare of participants and preserving public trust in the research institution. Furthermore, the pragmatism of a trial does not hinge on a single design domain. In cases where, for example, the pragmatism of trial recruitment is reduced by informed consent processes, researchers can look for ways to increase the pragmatism of other domains.

## Conclusion

Some argue that ethical protections can be in tension with trial pragmatism. This has even been used to argue against ethical protections in trials with pragmatic intent. But the relationship between pragmatism and ethical protections is more complex. Being mindful of the multidimensional nature of pragmatism and the potential for ethical protections to promote pragmatism could help investigators design trials that inform clinical and policy decisions while simultaneously safeguarding the rights and welfare of participants.
